# Effect of medication therapy management services on medication-burden quality of life in hemodialysis patients

**DOI:** 10.1186/s12882-023-03332-w

**Published:** 2023-09-20

**Authors:** Yasmine Salah Naga, Noha Alaa Hamdy, Amany El Bassiouny, Mohamed Selim, Samar Samy Abd ElHafeez

**Affiliations:** 1 Internal Medicine department, Nephrology Unit, Faculty of Medicine, Alexandria University, Alexandria, Egypt; 2 Clinical Pharmacy and Pharmacy Practice department, Faculty of Pharmacy, Alexandria University, Alexandria, Egypt; 3 Clinical pharmacist, Al Moassat University Hospital, Alexandria University, Alexandria, Egypt; 4 Epidemiology Department, High Institute of Public Health, Alexandria University, Alexandria, Egypt

**Keywords:** Medication therapy management, Medication burden quality of life, Hemodialysis, Drug related problems, Patient reported outcomes

## Abstract

**Background:**

Hemodialysis (HD) patients commonly receive polypharmacy leading to increased likelihood of drug related problems (DRPs) and poor quality of life. Medication Therapy Management (MTM) services discover and resolve DRPs and may specifically improve Medication-burden Quality of life (MBQoL) in HD patients. We aimed to assess the effect of MTM services on DRPs and MBQoL among HD patients.

**Methods:**

A prospective pre-post study was conducted on 104 patients in an HD unit in Alexandria, Egypt. MBQoL was assessed at baseline and after three months of MTM sessions, using the Arabic, validated version of the Patient Reported Outcomes Measure of Pharmaceutical Therapy (PROMPT) questionnaire. Cohen’s d test and multiple linear regression were used to assess the effect size of MTM and the factors affecting MBQoL, respectively. DRPs, adverse events and adherence were also monitored.

**Results:**

MBQoL improved significantly after the implementation of MTM (Cohen’s d=0.88, *p <* 0.01) with the largest effect size in the “medicine information and relation with healthcare providers” domain. DRPs decreased significantly after MTM implementation (11.97 ± 4.65 versus 7.63 ± 3.85 per patient, *p<*0.001). The mean adverse events per patient were also reduced (9.69 ± 4.12 versus 6.56 ± 3.77, *p <* 0.001).

**Conclusion:**

Applying MTM services presents an opportunity to improve care for HD patients by improving MBQoL, decreasing DRPs and adverse events.

## Background

Worldwide, over five million patients with end-stage renal disease (ESRD) are maintained on hemodialysis (HD), with more than 50,000 in Egypt [1, 2]. Patients on maintenance HD face multiple problems including high morbidity and mortality [3]; a financial burden on patients and health care systems due to the high cost of dialysis, medications, loss of productivity; and poor health-related quality of life (HRQoL) [4, 5].

Polypharmacy is common in HD patients to manage complications and co-morbidities; however, medications are often perceived as a burden leading to decreased patient adherence [6]. Poor adherence results in failure to achieve the therapeutic goals, prescribing unnecessary medications, ordering unneeded investigations, and thus wasting time and money [7]. Polypharmacy may also lead to drug related problems (DRPs), defined as events or circumstances involving drug therapy that actually or potentially interfere with the desired health outcomes. DRPs include improper drug selection, inappropriate dose or route of administration, adverse effects, drug interactions, failure to receive medication, an untreated indication and duplicate or unnecessary medications [8–10].

Thus, there is a growing need for continuous medication review among HD patients to avoid DRPs and improve adherence. This role can be fulfilled by the clinical pharmacist as part of a multi-disciplinary team in the form of medication therapy management (MTM) [11]. MTM is composed of five core elements, namely medication therapy review (MTR), personal medication record (PMR), medication-related action plan (MAP), intervention and/or referral, and, lastly, documentation and follow-up [8, 9, 11]. An update of MTM expanded its use to all patient care settings and added patient preferences and medication experience to further refine the treatment plan [9]*.* The potential role of MTM in HD patients is increasingly recognized with various efforts to standardize and widely implement it [12, 13].

MTM has a positive impact on chronic kidney disease [14] as well as HD patients by reducing hospitalization [15] and improving HRQoL [16–18]. Its benefit on HRQoL is also proven in other disease states, yet most studies use generic or disease-specific tools which are neither sensitive nor specific to changes induced by MTM interventions as they focus on the burden imposed by the disease. A systematic review including 48 MTM studies examined the effect of pharmaceutical care on HRQoL in various settings. Out of 1019 items in the used HRQoL tools, only 34 were specifically related to medicines and even those were not specific to the burden of medicine on functioning and well-being. Generic tools only show moderate sensitivity to pharmaceutical care, while disease-specific tools are not affected by MTM [19, 20].

Therefore, new tools were developed to specifically assess medication-burden quality of life (MBQoL) [21–24]. These include factors such as medication cost, drug-induced limitation of social or functional role, worries of adverse effects, and drug-drug interactions as possible causes for impaired quality of life [19, 22]. The patient-reported-outcomes measure of pharmaceutical therapy (PROMPT) scale is one of these tools, designed to identify drug related needs and to assess MTM interventions [24].

The impact of MTM on MBQoL has not been assessed in HD patients, therefor our aim was to evaluate the impact of providing MTM on MBQoL and its predictors among hemodialysis patients in an HD unit in Alexandria, Egypt using the PROMPT questionnaire. As a secondary outcome, we also assessed the impact of MTM on the number of DRPs, number of administered medications, adherence, adverse effects and laboratory parameters, which are all factors affected by polypharmacy and are expected to adversely affect MBQoL.

## Methods

### Study setting and design

This prospective pre-post intervention study was conducted in the HD unit of Almoassat University Hospital, Alexandria, Egypt, which is the largest dialysis unit in Alexandria and is a tertiary referral unit that includes patients with multiple co-morbidities. MBQoL was considered as a primary outcome for sample size calculations. The study was conducted after approval from the Ethics Committee of the High Institute of Public Health and in accordance with the 1964 Declaration of Helsinki. It started in January 2020 and extended to January 2021.

### Study population

**Fig. 1:**
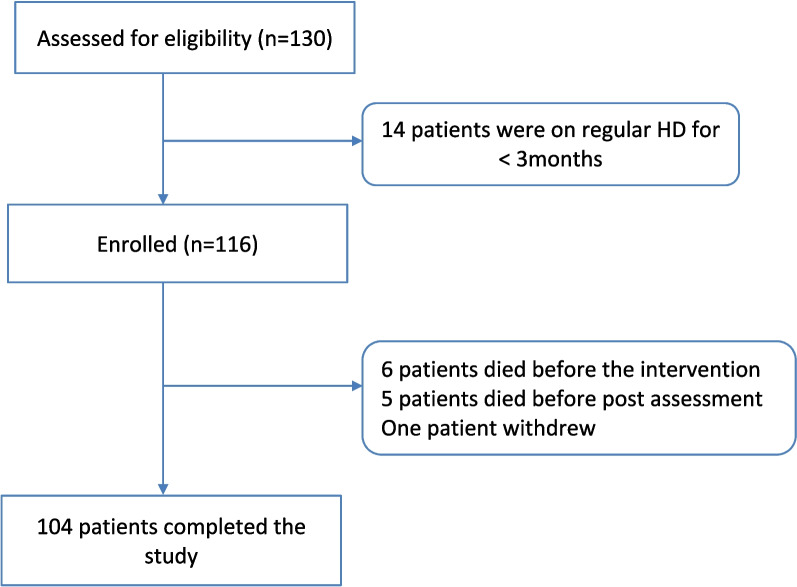
Flow chart of the study participants

The sample was calculated using R software version i 386, 3.6.0, based on an effect size of 0.3 [25], 5% alpha error, 0.80 power. The minimum sample required was 90 hemodialysis patients. The study included all patients in the hemodialysis unit who were older than 18 years and were on maintenance HD for more than 3 months. Of the 130 patients at Almoassat hemodialysis unit, fourteen patients were not eligible (on regular HD for < 3 months), one patient withdrew, eleven patients were lost to follow up (6 died before intervention, 5 died after intervention and before the post-assessment), and 104 patients completed the post-assessment interview (Fig. 1). The patients were included after obtaining an informed consent. In illiterate patients, the consent was read to them in the presence of a literate relative and they provided a fingerprint on the consent, which indicated their informed consent to participate. Each patient was monitored for three months to assess the baseline data, MTM interventions were applied and continued for another three months. After that, post-intervention assessment was performed.

### Data collection tools

A predesigned structured interview questionnaire was used to collect demographic and baseline data. It included the following sections: personal data including age, gender, marital status, education, working status and smoking; HD-related data including vascular access; co-morbidities; and administered medications. Baseline laboratory values related to anemia (hemoglobin level) and chronic kidney disease-related mineral bone disease (calcium, phosphorus and calcium phosphorus product) were recorded, as both are common complications in HD patients that are targeted by prescribed medications.

The PROMPT questionnaire was chosen to assess MBQoL as in comparison to other tools, it only requires around 5 minutes to fulfill, making it more practical. It also helps in identifying patient knowledge gaps regarding his medication to address them in the MTM intervention. The PROMPT was validated on a large number of subjects with different underlying disease and is responsive to changes made by MTM, which was our main concern [24].

After Arabic translation and cultural adaptation of the PROMPT questionnaire according to the guidelines, the psychometric properties were examined in another HD center before being used in our study. The Arabic version revealed good content validity and face validity. Convergent and divergent validity of the Arabic version were also proved. Results of internal consistency using Cronbach’s alpha (0.76) and test-retest reliability using intraclass correlation coefficient (ICC) revealed that the Arabic version of the questionnaire was reliable (under review for publication).

The PROMPT questionnaire consists of 16 items distributed in five domains. Each item has a 5-point Likert scale. The PROMPT total score is the sum of all 16 items, with the higher score indicating better QoL. Individual domain scores were transformed to range from 0 to 100 as follows: Domain score = 100 * (observed score − minimum domain score)/ (maximum domain score − minimum domain score). Interpretations of each domain score was 0 – < 25 low. 25- < 50 = fair to moderate, 50- < 75 = moderate to good, and 75 - 100 = good to excellent.

Each patient was asked to bring his medications to identify DRPs which were categorized according to Hepler-Strand classification [8]. Hepler and Strand, two of the pioneers of clinical pharmacy services proposed a classification for DRPs which is still commonly in use [25]. Hepler classification includes untreated indication, improper drug selection, subtherapeutic dose, failure to receive drug, overdose, adverse effects, and drug without indication [8]. Two items were added to this classification namely, duplicate therapy and improper administration timing to express the administration of two medications with the same active ingredient and the administration of the proper drug but at improper timing regarding meals, daytime, or hemodialysis, respectively.

Adherence was assessed for drugs to treat anemia (erythropoiesis stimulating agents (ESAs) and iron therapy), drugs for mineral bone disease (MBD) (calcium supplements, phosphate binders, and vitamin D analogs), and antihypertensive drugs by comparing the doses administered by patients to those prescribed by the physician. Total adherence represented the sum adherence of the three classes. If the patient was taking ≥ 80% of his doses, the patient was considered adherent [26].

Lastly, adverse effects were identified using the 'Patient-Reported Outcome Measure, Inquiry into Side Effects' (PROMISE) instrument checklist of 22 symptoms which included: “change of appetite, dry mouth/ thirst, nausea/ vomiting, stomach pain/ dyspepsia, abdominal pain, diarrhea, constipation, flatulence, eye irritation/ vision problems, palpitations, trembling/ shivering, muscle/ joint pain, muscular weakness, headache, dizziness/ vertigo/ fainting, weakness/ tenderness, drowsiness, change of mood, sexual complaints, bruises/ bleedings, skin complaints/ itching, and sweating”, a comprehensive checklist that has been validated in a large population of various patients. This checklist was previously used to identify and address adverse effects through pharmacist-based intervention and was suitable for our research [27, 28].

### First phase: assessment of baseline data

Baseline data were collected over a period of three months. During that period, patients continued to receive the usual medical care that was provided by the physician and nurses, in addition to simple advice that was given by the pharmacist during monthly dispensing of medications. Medication burden quality of life, DRPs, adherence and adverse effects were also assessed using the previously mentioned tools. Laboratory data were recorded from patient recorded.

To construct good relations with the study participants, the data collector, who is a certified clinical pharmacist, started to show up in the dispensing pharmacy and introduced herself to the patients during the dispensing process. She started short conversations with the patients and gave them an idea about the aim of the research. A detailed explanation of the study procedure was performed. Those who agreed to be part of this study were included in the study. Data were collected from patients at their bedsides. If the patient was unable to communicate due to fatigue of any cause or if he was not ready to communicate, this was respected and told that the data collection will be postponed until he is ready.

### Second phase: intervention phase 

During the second three months, MTM interventions were applied including performing a medication review, participating in the treatment plan by selecting, modifying, or administering medication therapy, evaluating the patient response to therapy, identifying, and resolving any DRPs, and providing patient education to enhance the patient understanding and adherence to the treatment plan and to encourage self-management strategies [29, 30]. Educational sessions were provided to patients at least monthly. The first session lasted 30 to 60 minutes while follow up sessions were 5 to 15 minutes long. During these sessions, the treatment plan was explained, as well as the indication of each medication, dose, appropriate administration route and time, and what to do if a dose was missed.

Some of the misconceptions that needed to be clarified were the proper timing of phosphate binders within meals, the purpose and indication of iron and erythropoiesis stimulating agents (ESA) in the treatment of anemia, proper use of vitamin D derivatives, safe analgesics and proper doses of non-steroidal anti-inflammatory drugs (NSAIDs) when indicated. Duplicate therapy was rectified. Possible adverse effects due to medications or HD complications and their prevention were addressed. Instructions for appropriate nutrition and care of HD access were also given. Causes of poor adherence were identified and resolved.

Patients welcomed the education process and continue to consult and seek education regarding their medications and any suspected adverse event from the unit clinical pharmacists till now.

### Third phase: post-intervention assessment

At the end of the intervention period, participants were reassessed for MBQoL, DRPs, adherence, adverse effects and laboratory parameters using the same tools.

### Statistical analysis

Data were summarized using mean ± standard deviation (SD) for continuous normally distributed variables, median and interquartile range (IQR) for continuous non-normally distributed variables, and frequency and percentage for categorical variables. Data were compared using paired t-test or Wilcoxon signed rank test according to the normality distribution. In addition, Cohen’s d-test was used to quantify the magnitude of difference (effect size) of the PROMPT total and domain scores. The effect size was classified according to Cohen’s d value as negligible (< 0.2), small (0.2- <0.5), moderate (0.5- <0.8), and large (≥0.8). Multiple linear regression analysis was used to identify the predictors of the PROMPT score after MTM implementation. Variables with *p*-value <0.1 in the bivariate analysis were included in the model [31]. The final model included the following variables: university graduates or higher, working status, smoking, hospital admission post-MTM implementation, having a fistula as HD access, adherence to antihypertensives, and the total DRPs after MTM implementation. SPSS version 21 was used to analyze the data and two tailed *p*-value <0.05 was considered significant.

## Results

### Patients’ characteristics

Table 1 displays the characteristics of the study population. Among the 104 HD patients, the mean age was 51 ±12 years, 50% were males, 80.8% were married, 24% were employed, and 22.2% were university graduates or higher. 60.6% had cardiovascular diseases, 24% had liver diseases, and 15.4% were diabetics. Most patients (92.3%) were on a thrice weekly dialysis schedule, with a median (IQR) of dialysis vintage 5 (2-14) years and 84.6% had arteriovenous fistulas as their vascular access.

**Table 1:** Baseline characteristics of the study population

Characteristics	*N=*104 (%)
Age (years)	51±12.32
Male (%)	52 (50)
Marital Status (%)	
Single	17 (16.3)
Married	84 (80.8)
Divorced	2 (1.9)
Widowed	1 (1)
Education (%)	
Illiterate	10 (9.6)
Basic Education	28 (26.9)
Secondary "Moderate"	43 (41.3)
University or Higher	23 (22.2)
Smokers (%)	27 (26)
Employed patients (%)	25 (24)
Co-morbidity (%)	
Cardiovascular diseases	63 (60.6)
Liver disease	25 (24)
Diabetes mellitus	16 (15.4)
Respiratory disease	12 (11.5)
Mineral bone disease	6 (5.8)
Gastro-intestinal disease	5 (4.8)
Rheumatic disease	4 (3.8)
Others^a^	11 (10.6)
Dialysis Vintage (years)	5 (2-14)
Dialysis sessions per week	
Twice	8 (7.7)
Thrice	96 (92.3)
HD access (%)	
Fistula	88 (84.6)
Graft	2 (1.9)
Catheter	14 (13.5)

*SD* Standard deviation

^a^Include thyroid dysfunction, eye disease, skin disease and mental illness

### Medication burden quality of life (MBQoL)

At baseline, 40 patients (38.5%) had fair to moderate MBQol and 62 (59.6%) had moderate to good MBQol. The mean total PROMPT score was 50.74±9.68. The lowest MBQoL score was in the “medicine information and relation with health care providers” domain (45.43±17.66) and the highest in the “administration-related concern” and the “medicine problems concern” domains (63.46±29.43 and 65.26±30.96, respectively) (Table 2).

**Table 2:** A comparison of participants' response to the PROMPT questionnaire pre- and post MTM-implementation

PROMPT items	Pre MTM	Post MTM	*P*-value
	Median (IQR)		
Domain 1: Medicine information and relation with health care providers			
Indications for treating your diseases or relieving the symptoms			
Proper use of medicines	4(2-4)	5(5-5)	<0.001^*^
What to do if you missed medicine doses	4(3-4)	5(5-5)	<0.001^*^
Side effects possibly caused by your medicines and resolving them	1(1-1)	2(1-4)	<0.001^*^
Symptoms, severity, and treatment of the disease	1(1-1)	1(1-3)	<0.001^*^
Think the doctor, pharmacist, or nurse have friendly manners and give you an opportunity to ask questions about your medicines?	4(2-4)	5(4-5)	<0.001^*^
	4(3-5)	5(3-5)	0.14
Domain 1 total score (Mean± SD)	45.43±17.66	66.87±17.48	<0.001^*^
	Cohen’s d =1.23		
Domain 2: Medicine effectiveness			
Alleviating the symptoms	3(2-4)	4(3-4)	0.01^*^
Curing the disease	3(2-4)	3(2-3)	0.04^*^
Domain 2 total score (Mean± SD)	52.04±30.57	52.40±28.09	0.92
	Cohen’s d =0.01		
Domain 3: Administration related concern			
Feeling bored or uncomfortable for using your medicines every day	4(2-5)	5(3-5)	0.003^*^
Medicine dependance	4(2-5)	5(3-5)	0.06
Ease of use	4(3-5)	5(4-5)	<0.001^*^
Domain 3 total score (Mean± SD)	63.46±29.43	75.72±28.16	<0.001^*^
	Cohen’s d = 0.43		
Domain 4: Medicine problems concern			
Medicines interacting with each other	5(3-5)	5(3-5)	0.15
Adverse drug effects	4(2-5)	5(4-5)	<0.001^*^
Domain 4 total score (Mean± SD)	65.26±30.96	78.73±24.50	<0.001^*^
	Cohen’s d = 0.48		
Domain 5: Impact on patient’s life			
Working, study, household chores, hobbies, or socializing with friends or relatives			
Medicine and travel expenses	4(3-5)	5(3-5)	0.11
Overall, how do your medicines improve your life?	3(2-5)	3(2-5)	0.1
	3(3-4)	3(3-4)	0.02^*^
Domain 5 total score (Mean± SD)	56.97±21.45	64.02±24.00	0.004^*^
	Cohen’s d = 0.31		
Overall PROMPT score	50.74±9.68	59.3± 9.86	<0.001^*^
	Cohen’s d = 0.88		

*IQR* Interquartile range, *SD* standard deviation

^*^Significant (*p<*0.05)

**Fig. 2:**
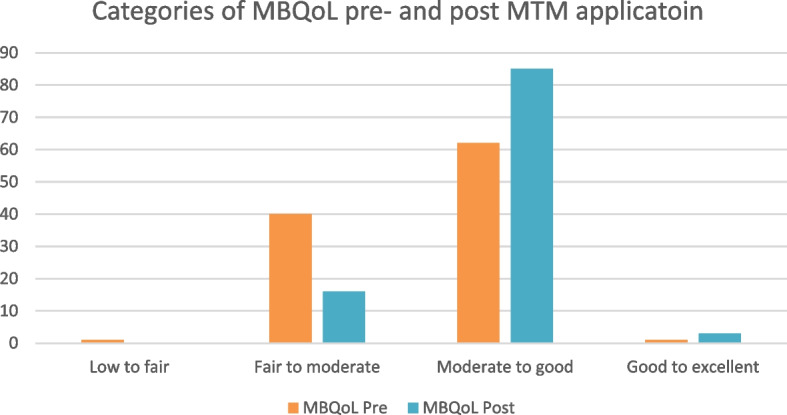
MBQoL before and after intervention

After the intervention, a statistically significant increase was reported in the total PROMPT score with a moderate effect size (0.88, *p<*0.001) (Table 2), with the largest effect size in the “medicine information and relation with health care providers” (1.23, *p<*0.001). The number of patients with moderate to good MBQoL increased from 62 (59.6%) to 85 (81.7%) (Fig. 2).

### Drug related problems

The most common drug related problems before and after MTM implementation were adverse effects and untreated indication. The total number of DRPs per patient decreased significantly from 11.97**±**4.65 to 7.63**±**3.85 after MTM implementation (*p<*0.001). There was a statistically significant reduction in six categories of DRPs: adverse effects (*p<*0.001), subtherapeutic dose (*p=*0.001), failure to receive drugs (*p=*0.004), drug overdose (*p=*0.002), drug interaction (*p=*0.001), and duplicate therapy (*p=*0.001) (Table 3).

**Table 3:** Comparison of DRPs pre- and post the implementation of MTM

	Pre MTM	Post MTM	
	No. of events (Percentage out of total number of DRP)		*p*-value
Untreated indication	56 (4.5%)	47 (5.91%)	0.43
Improper drug selection	22 (1.77%)	14 (1.76%)	0.14
Subtherapeutic dose	27 (2.17%)	9 (1.13%)	0.001*
Failure to receive drug	24 (1.93%)	7 (0.88%)	0.004*
Overdose	39 (3.13%)	16 (2.02%)	0.002*
Adverse effects	1008 (80.96%)	682 (85.89%)	<0.001*
Drug interaction	13 (1.04%)	0 (0%)	0.001*
Drug without indication	20 (1.61%)	10 (1.26%)	0.07
Mode of administration (Timing)	18 (1.45%)	9 (1.13%)	0.05
Duplicate therapy	18 (1.45%)	0 (0%)	0.001*
Total Number of drug related problems Mean± SD (DRPs/patient) **	(1245) 11.97±4.65	(794) 7.63±3.85	<0.001*

^*^Significant (*p<*0.05), **Using paired t-test, Wilcoxon-signed rank was used for the DRPs categories

### Drug adherence pre- and post-MTM implementation

Patients took on average 8 medications. There was no significant difference in the number of administered medications pre- and post-MTM implementation (8.10±2.78 versus 8.29±2.57, *p=* 0.28). The lowest rate of adherence was to MBD medications followed by anemia medications (53.9% and 56.9%, respectively). While the percentage of adherent patients improved in the three categories post-MTM, this increase was not statistically significant (Table 4).

**Table 4:** Comparison of the number of administered medications and drug adherence pre- and post-MTM implementation

	**Pre**		**Post**		***P*-value**
	**No.**	**%**	**No.**	**%**	
**Overall adherence percentage**	45	44.1	57	55.9	0.06
**Percentage of patients adherent to anemia medications (*n=*72)**	41	56.9	46	63.9	0.44
**Percentage of patients adherent to MBD medications (*n=*89)**	48	53.9	51	57.3	0.65
**Percentage of patients adherent to antihypertensive medications (*n=*64)**	56	87.5	58	90.6	0.69
**No. of administered medications (mean ±SD)**	8.10±2.78		8.29±2.57		0.28

*MBD* Mineral bone disease, *SD* standard deviation

^*^Significant (*p<*0.05)

### Adverse effects

**Fig. 3:**
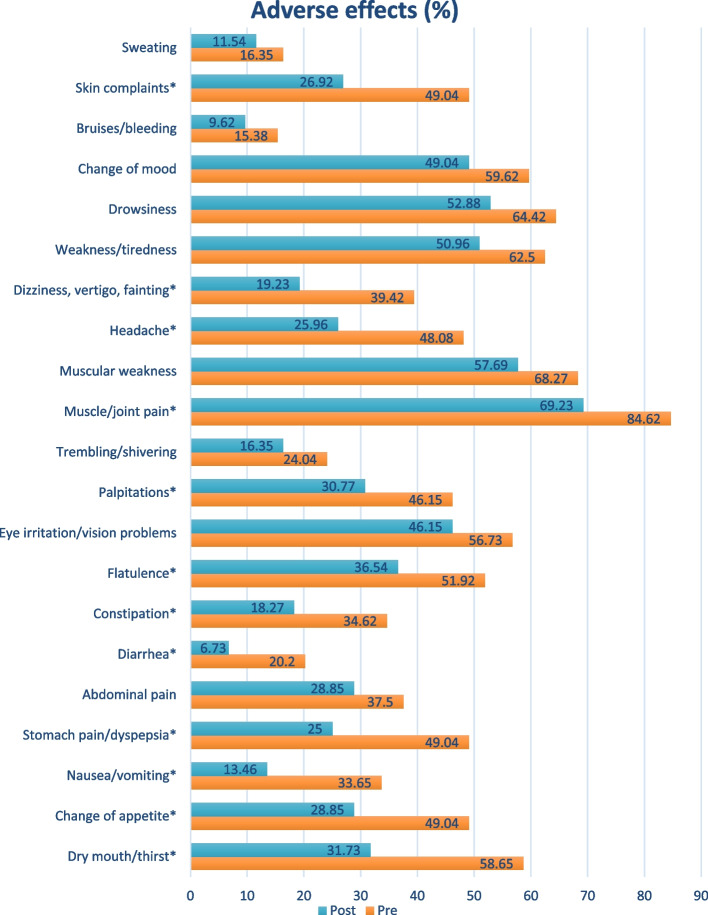
Comparison of adverse effects percentage pre- and post-MTM implementation. ^*^Significant (*p<*0.05)

The total number of adverse events decreased significantly from 1008 to 682 events (*p<*0.001) after the implementation of MTM. The most commonly reported adverse event was muscle/joint pain (84.6%), with more than 50% reporting muscular weakness (68.27%), drowsiness (64.42%), weakness/tiredness (62.50%), change of mood (59.62%), dry mouth/thirst (58.65%), eye irritation/vision problems (56.73%) and flatulence (51.92%). There was a statistically significant reduction from pre- to post-MTM implementation in the occurrence of dry mouth, change of appetite, nausea and vomiting, stomach pain and dyspepsia, diarrhea, constipation, and flatulence, palpitations, muscle and joint pain, headache, dizziness, vertigo, fainting, and skin complaints (Fig. 3 ).

### Laboratory parameters

There was a statistically significant decrease in serum calcium level (9.13±0.95 versus 8.77±1.05, *p=*0.002). The change in other reviewed laboratory parameters was not statistically significant (Table 5).

**Table 5:** Comparison of laboratory parameters pre- and post the application of MTM

	**Pre MTM**	**Post MTM**	***p*-value**
	**Mean± SD**		
**Hemoglobin g/dL (***n***=**85), target (10-11)	9.90±2.03	9.56±2.21	0.15
**Calcium mg/dL (*n=*86),** target (8.5-10.5)	9.13±0.95	8.77±1.05	0.002*
**Phosphorus mg/dL (*n=*86)** target (2.5-4.5)	5.74±1.79	5.57±1.70	0.33
**Calcium phosphorus product (*n=*79),** target <55	52.63 ±18.02	49.46±16.83	0.06

*Significant (*p*<0.05)

### Predictors of the MBQoL

Education, working status, and the proportion of patients who were adherent to antihypertensive medications were significantly correlated to the PROMPT score post MTM implementation, (r_s_= 0.25, *p=*0.01), (r_s_=0.24, *p=*0.02), and (*r*=0.31, *p=*0.01), respectively. A statistically significant inverse correlation was found between the total number of DRPs and the total PROMPT score post MTM implementation (*r* =-0.57, *p<*0.001).

The multiple linear regression analysis model explained 47% of the variation in the PROMPT score by the variation in education, and the total DRPs post MTM. In this model, being a university graduate or higher increased the score by 7.64 folds (*p=*0.004), and the presence of each DRP decreased the score by 1.49 folds, (*p<*0.001) (Table 6).

**Table 6:** A multiple linear regression model of the predictors of total PROMPT score post-MTM implementation

	**B**	***P*-value**	**95.0% Confidence Interval for B**	
			**Lower Bound**	**Upper Bound**
**(Constant)**	72.60	<0.001*	59.65	85.56
**University education or higher (versus secondary education or lower)**	7.64	0.004*	2.60	12.69
**Smoking (versus non-smoking)**	-3.33	0.16	-8.01	1.35
**Working status (working versus unemployed)**	-0.44	0.87	-5.70	4.81
**HD access (AVF versus other)**	-3.87	0.25	-10.58	2.84
**Hospital admission post-MTM**	4.50	0.25	-3.29	12.28
**Adherence to antihypertensives (adherent vs nonadherent)**	2.72	0.47	-4.76	10.20
**Total drug related problems post-MTM**	-1.49	<0.001*	-2.11	-0.88

^*^Significant (*p<*0.05)

## Discussion

As anticipated in our hypothesis, providing medication therapy management to hemodialysis patients in our study improved MBQoL as assessed by the PROMPT questionnaire, reduced DRPs and adverse events. Adherence and most laboratory parameters, however, did not improve significantly. At the end of the intervention, patients developed confidence, trust and more enthusiasm to be more engaged and more compliant with their treatment which was translated into these positive outcomes.

Improvements in the patients’ MBQoL was reflected by an increase in total PROMPT score after implementing MTM. The large effect size (0.88) in the total score was mainly driven by the improvement in the first domain, “medicine information and relation with health care providers” (1.23). A similar effect was reported by Sakthong and her colleague who used the PROMPT questionnaire to assess the impact of MTM on MBQoL in patients of a tertiary hospital. Their study included 514 patients with multiple comorbidities either randomized to receive pharmaceutical care or usual care. In the intervention group, a large effect size was noted in the total score (1.44) driven mainly by the improvement in “medicine and disease information domain” (3.23) [25].

Pharmacist interventions improve the patients’ knowledge and attitude toward their medications, improve adherence, relieve medication burden, and consequently improve their QoL as observed in previous studies for HD patients. Yet, previous studies used either general, such as the short-form 12 (SF-12); or disease-specific instruments such as SF-36, Kidney Disease Quality of Life-36 (KDQoL-36) and renal quality of life profile (RQLP) to assess the effect of MTM on HRQoL [16–18, 32].

Most recently, a study by Al-Mansouri et al. found a link between treatment burden and HRQoL as assessed by the KDQoL questionnaire in pre-dialysis and HD patients [33]. Treatment burden is defined as the burden imposed on a patient by his treatment plan and its effect on his QoL [34]. In their study, they used the treatment burden questionnaire that explores medication-induced, lifestyle change-induced, administrative, financial and social aspects of treatment. The highest treatment burden was medication and lifestyle-change burden; and higher treatment burden was associated with worse HRQoL [33].

University education or higher and post-MTM total DRPs were identified as the major contributors to the total MBQoL score after MTM implementation. Although multiple previous studies found an association between gender, age, marital status and HRQoL [16, 35–37], our study found no such effect, which may be attributed to our use of a more specific tool.

University graduates had higher PROMPT scores compared to lower educational levels. The association between higher education and better HRQoL was also observed in other studies [38–40]. Educated patients were more interactive during intervention sessions. Those patients were keener to understand their medical problem in detail, more concerned about adverse effects and drug interactions, and were seeking advice to avoid them, which was reflected in their knowledge and behavior, and reduced DRPs significantly. Although this should not deter the provision of health education to all patients regardless of their level of education, it should lead to a more tailored approach provided to each patient depending on his educational level.

In our study, DRPs were significantly reduced by MTM and their number post-MTM was a significant predictor of post-intervention MBQoL. There was also an inverse relation between the number of DRPs and MBQoL. The reduction of DRPs after MTM in our cohort is similar to that observed by other researchers [16, 25, 32]. Drug related problems were also predictors of MBQoL in the trial carried out by Sakthong *et al.* They found an improvement in overall DRPs and adverse effects profile as a result of MTM implementation, where improvement in the PROMPT score was associated with the number of DRPs that were resolved through MTM interventions [25]. The DRPs were resolved through identifying and filling the knowledge gaps that the patients had, addressing reversible adverse events as well as by reviewing and adjusting medication dosing, frequency, and timing.

At baseline, we reported 11.97 DRPs per patient which is higher than that found by Pai *et al* (8.6 DRP per patient) in dialysis patients [41]. The most common DRP found in our study was adverse effects (80.96%), followed by untreated indication (4.5%), drug overdose (3.13%), and subtherapeutic doses (2.17%). In contrast, a systematic review summarizing pharmacist activities in ESRD patients found untreated indication to be the most common DRP followed by subtherapeutic or supratherapeutic dose, and medical record discrepancies [42]. Different studies, however, use different DRP assessment tools making the comparison across studies difficult and most tools are not adequately validated [43]. Also, use of the term “treatment-related problems” instead of “drug-related problems” has been suggested to include untreated indications, which is not directly “drug-related” [44]. This change may be especially beneficial in the HD population to include dialysis-related problems.

A recent retrospective study reviewed clinical pharmacist care provided in 14 dialysis centers across Southeast Michigan, USA. They found an average of 8.96 medication-related problems per patient with adherence being the most common followed by the need for additional drugs. Beside a potential cost avoidance by reduction in physician visits and hospitalizations, they observed an increase in patients within target levels of blood pressure and MBD markers. Although they did not assess MBQoL, they conducted a post-medication reconciliation follow-up patient survey, in which 94.7% of responders reported that pharmacists helped them understand their medications and 77% reported better adherence [45].

Another retrospective study examined the effect of multi-disciplinary MTM provided by a team of nurses, pharmacists and nephrologists to HD patients on hospital discharge from acute care hospitals. They found that improper dosing, adverse drug reaction(s) and unnecessary drug therapy were the most common medication related problems. Compared to patients who did not receive MTM on discharge, full MTM significantly decreased the risk of 30-day rehospitalization [46].

Our detection of more DRPs and why adverse events were the most common DRP are probably attributed to our use of an adverse effects checklist to capture all adverse effects related to medications or related to HD. Adverse effects are usually under-reported as noted in a systematic review of MTM studies in CKD patients [47]. Yet, some of the adverse effects in the list used may not be related to medications or dialysis but rather to diet or underlying co-morbidities. Still, their improvement after MTM implementation and patient education was significant. Examples of resolved adverse events by our MTM intervention included the following: dry mouth was due to excessive salt intake in between HD sessions, nausea, vomiting, headache, dizziness, and palpitations were either due to intradialytic hypotension or due to administration of alpha-receptor blockers, and all improved by addressing the corresponding issues. Skin complaints and itching due to hyperphosphatemia or dialysis inadequacy, improved on instructing patients on taking their phosphate binders within meals and the importance of completing their dialysis session. Similarly, a reduction in adverse effects as a result of MTM implementation among HD patients was reported by Dashti [32].

Adherence is another important outcome of MTM. It is affected by regimen complexity, number of pills administered, adverse effects, medication cost, not involving the patients in the treatment decision and patients’ beliefs [29, 48, 49]. In our study, the pharmacist tried to improve the patients’ knowledge regarding medications, engaging the patients in the treatment decisions, and overcoming adverse effects induced by medications. Medications without indication were identified and discontinued. Adherence improved but the improvement was not statistically significant, unlike in some MTM studies [17, 42, 47].

The laboratory parameters recorded before and after intervention did not significantly improve, which may be because of the short follow-up period. Some studies also failed to observe an improvement in laboratory parameters like hemoglobin concentration, which was not improved in the study by Pai et al [16], while others reported improvements in hemoglobin in response to MTM applications [47, 50].

The current study was conducted largely during the first year of the COVID-19 pandemic. The pandemic affected HD patients negatively in many ways. Patients receiving maintenance HD are more susceptible to COVID-19 infection due to their impaired immune system, there was a need for attending health care facilities three times per week and being in close contact with other patients and with healthcare providers for about 4 hours each dialysis session even during periods of lockdown [51]. During lockdown, it was difficult to maintain physical activity or to stick to dietary restrictions. Transport from and to dialysis centers was another challenge leading to missed dialysis sessions [52, 53]. There was also a shortage of personal protective equipment, and of some imported medications [54, 55]. Despite these factors, an improvement in MBQoL was achieved by MTM. The patients continue to seek advice from the unit clinical pharmacist with recruitment of more clinical pharmacist in our unit and in other sections of the hospital. MTM should be part of standard patients care in HD units due to its beneficial impact on MBQoL, DRPs and adverse events.

Our study has several strengths. It is the first study to specifically assess the effect of MTM on MBQoL in HD patients. We used a novel and specific tool, the PROMPT questionnaire, while previous studies assessed HRQoL using generic and disease-specific tools. It was carried out in a tertiary health facility that possesses the largest HD unit in Alexandria, Egypt and that includes HD patients with multiple co-morbidities. Our sample was adequate to detect a significant difference in the primary outcome. We also used an adverse effects checklist to overcome the problem of underreporting. Moreover, we observed an improvement in MBQoL despite the COVID-19 pandemic.

Limitations of the study include the absence of a control group, the relatively short follow-up period and the COVID pandemic, which may have attenuated the benefit of MTM on MBQoL. Another limitation of the current study is the relatively high attrition rate, which is common in HD populations. However, this was accounted for during sample size calculation, therefore, it did not affect the results. The presence of multiple alternative tools to assess drug/treatment-related problems, MTM and MBQoL with absence of consensus on the ideal instrument is another limitation of the study. Future efforts should aim at comparison of different instruments and identification of the best one for different patient categories.

## Conclusion

Medication-burden is significant in HD patients. Therefore, medication therapy management should be an essential part of a comprehensive multi-disciplinary care of HD patients. Implementation of MTM to HD patients resolved drug-related problems and adverse effects and led to improved medication-burden quality of life despite the COVID-19 pandemic. Using a specific tool for assessing humanistic outcomes of MTM implementation is recommended to accurately assess their impact on patient care. Our findings have wider implication in chronic diseases rather than in acute illnesses. MTM should be an important component of chronic patient care and its effect on MBQoL should be studied in other chronic disease states managed by polypharmacy. MTM intervention and MBQoL tools should be customized appropriately in each setting to address disease-specific factors.

## Data Availability

All datasets used and/or analyzed are available from the corresponding author on reasonable request.

## Abbreviations

CKD: Chronic kidney disease
COVID-19: Coronavirus disease 2019
DRPs: Drug related problems
ESAs: Erythropoiesis stimulating agents
ESRD: End-stage renal disease
HD: Hemodialysis
HRQoL: Health-related quality of life
IQR: interquartile range
KDQoL-36: Kidney Disease Quality of Life-36
MAP: Medication-related action plan
MBD: Mineral bone disease
MBQoL: Medication-burden Quality of life
MTM: Medication Therapy Management
MTR: Medication therapy review
NSAIDs: Non-steroidal anti-inflammatory drugs
PMR: Personal medication record
PROMISE: Patient-Reported Outcome Measure, Inquiry into Side Effects
PROMPT: Patient-reported-outcomes measure of pharmaceutical therapy
PROMPT-QoL: Patient-reported-outcomes measure of pharmaceutical therapy-quality of life
QoL: Quality of life
RQLP: Renal quality of life profile
SD: Standard deviation
SF: Short form
